# Occupational practice in patients with hereditary transthyretin amyloidosis, a qualitative study

**DOI:** 10.1186/s13023-023-02964-3

**Published:** 2023-11-10

**Authors:** Aina Gayà-Barroso, Juan González-Moreno, Adrián Rodríguez, Tomás Ripoll-Vera, Inés Losada-López, Margarita Gili, Milena Paneque, Sara Pérez-Martínez, Eugenia Cisneros-Barroso

**Affiliations:** 1 Internal Medicine Department, Son Llàtzer University Hospital, Palma, Spain; 2 Health Research Institute of the Balearic Islands (IdISBa), Son Llàtzer University Hospital, Palma, Spain; 3 Cardiology Department, Son Llàtzer University Hospital, Palma, Spain; 4 Department of Psychology, University of Balearic Islands, Spain. Health Research Institute of the Balearic Islands (IdISBa), Palma, Spain; 5 Institute for Research and Innovation in Health (i3S), University of Porto, Porto, Portugal; 6 Center for Predictive and Preventive Genetics, Institute for Molecular and Cell Biology (CGPP-IBMC), Porto, Portugal; 7 Institute of Biomedical Sciences Abel Salazar, University of Porto, Porto, Portugal; 8 Departament of Experimental Psychology, Cognitive Processes and Speech Therapy, University Complutense de Madrid, Madrid, Spain

**Keywords:** Activities of daily living (ADL), Hereditary transthyretin amyloidosis, Occupational therapy, Quality of life, Rare genetic disease

## Abstract

**Background:**

Hereditary transthyretin amyloidosis (ATTRv) is a rare genetic disease that negatively affects patients' quality of life through the involvement of various organs and tissues. Despite a large amount of research on medical and psychosocial interventions, the impact of occupational therapy (OT) on patients with ATTRv is not well understood.

**Objective:**

The aim of this study was to develop an OT programme to improve the daily functioning and quality of life of patients with ATTRv.

**Methods:**

Fourteen patients with ATTRv were interviewed. Together they developed short- and medium-term occupational goals. Patients received the OT intervention for six months. Outcomes were measured using scores for activities of daily living and psychological well-being.

**Results:**

The study found that OT can have a positive impact as a complementary intervention to medical and other psychosocial treatments. Of the 14 patients, 12 maintained the same scores in activities of daily living. Two deteriorated and eight improved their psychological scores.

**Conclusion:**

This study highlights the need for further research in this area and the importance of OT in the management of patients with ATTRv. Early intervention is of paramount importance and further research is needed to evaluate the long-term effects of OT interventions in patients with ATTRv.

**Supplementary Information:**

The online version contains supplementary material available at 10.1186/s13023-023-02964-3.

## Introduction

Rare diseases are defined as diseases that affect a small proportion of the population and that do not receive the same attention to treat and research as more common diseases [1–4]. Over 7000 rare diseases have been identified in Europe, including hereditary transthyretin amyloidosis (ATTRv) [5, 6]. ATTRv is caused by a mutation in the gene encoding transthyretin (TTR). It is characterised by the deposition of amyloid in multiple organs and tissues, leading to their dysfunction [7–11]. Within Spain, Valverde del Camino (Huelva) and Mallorca (Balearic Islands) are considered the main endemic foci for ATTRv, with an estimated prevalence of approximately 11 cases per 100,000 inhabitants in Mallorca. ATTR Val50Met represents a diagnostic challenge due to its clinical manifestations and multiorgan involvement, with significant variability in signs and symptoms documented between patients and between geographical regions. Sensorimotor polyneuropathy, autonomic dysfunction, cardiac, renal, ocular and gastrointestinal dysfunction have been described as the main disease manifestations associated with amyloid deposition [11–13]. The natural history of ATTRv with polyneuropathy has traditionally been defined using the Coutinho [14] and Yamamoto [15] scales. Coutinho et al. described the disease in 3 stages: Stage 1—The disease is limited to the lower limbs. Slight weakness of the extensor muscles of the big toes.

Stage 2—Motor signs progress in the lower limbs with step and distal amyotrophies, the muscles of the hands begin to become wasted and weak. The patient is obviously disabled, but can still get around, although with help. Stage 3—The patient is bedridden or wheelchair-bound, with generalised weakness and areflexia. On the other hand, Yamamoto and colleagues published a new description of the severity of the disease as follows PND I—sensory disturbances in the extremities with preserved ability to walk, PND II—difficulty walking but without the need for a cane, PND IIIa—one cane or crutch required for walking, PND IIIb—two canes or two crutches required for walking, and PND IV—patient confined to a wheelchair or bed.

As ATTRv typically manifests itself in adulthood, at a crucial time of occupational change, its onset and progression can have a significant impact on the lifestyle, daily activities and general well-being of patients, as well as on their families and carers [11–13, 16]. Physical, mental, occupational and social limitations are experienced by patients with this diagnosis and their carers. Furthermore, the psychological, occupational and social consequences of each disease stage are not fully described [11–13].

The pharmacological treatment options for ATTRv include treatments that stabilise TTR or treatments that block the genetic material that is responsible for the production of transthyretin in the cells [17]. However, there is currently no literature describing interventions with patients once they have been diagnosed and are receiving medical treatment in the psychosocial field and occupational therapy (OT) in particular. This represents a critical issue, as treatments improve and patients live longer, but managing daily life and mental health may not.

As a health care discipline, OT focuses on the promotion of health and well-being through everyday activities. The role of occupational therapists is to intervene and enable patients with rare diseases such as ATTRv to develop, regain, eliminate or compensate for the skills needed to successfully perform meaningful activities when there are occupational limitations, whether physical, psychological, social or environmental in origin [18–24].

As such, the field of psychosocial disciplines, and OT in particular, should develop and respond to the needs of patients, multidisciplinary teams and healthcare systems, as our identities are based on our occupations, routines and roles [12]. Recent studies in other conditions, such as Charcot-Marie-Tooth (CMT), multiple sclerosis and Parkinson's disease, show that people who have received or are receiving OT services are receiving them too late in the disease process. This has a negative impact on the effectiveness of occupational practice. This lack of knowledge about the profile of occupational therapists and the time elapsed between diagnosis and the start of occupational interventions may also have an impact on the effectiveness of occupational interventions for ATTRv patients [12, 13, 19].

A six-month occupational intervention was carried out with fourteen patients diagnosed with ATTRv, including 4 (28.6%) males and 10 (71.4%) females, with an average age of 57 years. Most of them had Coutinho stage I polyneuropathy and were independent in their daily activities. In order to obtain the parameters on which the work of the occupational therapist could have an impact, initial and final assessments were carried out using the Barthel Index (BI) and Brody Instrumental Activities of Daily Living Scale (IADL) to measure activities of daily living. The SF-36 spanish version V2 questionnaire and the Warwick-Edinburgh Mental Well-Being Scale (WEMWBS), in order to observe mental health parameters, and the Norfolk Quality of Life-Diabetic Neuropathy (Norfolk QoL-DN) questionnaire, validated in the ATTRv population, in order to verify common ATTRv symptoms and physical status [25–33]. In view of the lack of standardised occupational scales, open-ended questions have been developed relating to activities of daily living and autonomy [34–37].

The aim of this study is therefore to describe the occupational intervention applied to patients enrolled in the OT project and to determine the effectiveness of the occupational therapist intervention by means of standardised scales and a semi-structured interview.

## Methods

### Study design

This was an experimental study that aimed to evaluate the effectiveness of an occupational intervention on the activities of daily living of patients with ATTRv.

### Study population

Patients who were eighteen years of age or older and had a diagnosis of ATTRv were eligible for the study. Participants were recruited through the database of the Son Llàtzer University Hospital and through the web pages of patient support groups. Individuals who expressed interest in the study and met the inclusion criteria (i.e. association with patient support groups and commitment to attend sessions) were enrolled in the programme for a period of six months after the initial semi-structured interview. Patients who were interested in participating in the study but not in the interventions underwent semi-structured interviews at the beginning and end of the project.

### OT and ATTRv approach

The OT intervention was designed according to the Model of Human Occupation (MOHO) [38]. The full description of the intervention design is provided in the Additional file 1: Supplementary Information.

### Data collection

A semi-structured baseline interview was carried out between the 1st of September and the 10th of October 2021, and the data collected from this interview have been published in [19]. After 6 months, from the 10th of April to the 1st of May 2022, the same questionnaires were administered to the initial cohort of forty-four patients. In order to identify any changes or improvements, the results were then compared and analysed.

### Scales

The scales and questionnaires used in this work have been described in detail in [19, 25–37]. A brief description of the scales and questionnaires used in this work can be found in Additional file: 2 Supplementary Table.

### Statistical analysis

In this study, statistical analysis was carried out using the Statistical Package for the Social Sciences (SPSS), version 23 to examine the data. Descriptive statistics, including means, standard deviations, and frequencies, were calculated to provide a summary of key variables. To assess the differences among groups, an analysis of variance (ANOVA) was employed. Specifically, a one-way ANOVA was conducted to investigate the effects of the Occupational intervention on Barthel Index (BI) and Brody Instrumental Activities of Daily Living (IADL).

### Ethical considerations

This study has received the ethical approval of the Ethics Committee of the Balearic Islands and of the Research Commission of the Hospital Universitario Son Llàtzer (decision number: IB 4587/21 PI). All subjects were required to sign an informed consent form before any study-related procedures were initiated. This was done regardless of whether they agreed to participate in the six-month intervention programme.

## Results

A total of forty-four patients were enrolled in the occupational therapy (OT) project between 2021 and 2022. Of these, thirty (68.18%) were women. The mean current age was 53.06 (± SD = 13.9) years. The mean age at onset was 43.51 (± SD = 16.3) years. Of the forty-four patients, only fourteen (32%) were willing to take part in the occupational programme. Among the included patients, eleven (78.5%) were in Coutinho stage I polyneuropathy and independent in daily activities, two (14.3%) were in stage II with few difficulties in daily activities and one (7.1%) was in stage III and dependent. Thirteen (93%) of the enrolled patients were receiving medical treatment. Eight (57.1%) were receiving patisiran, five (35.7%) were receiving tafamidis and one (7.1%) was not receiving any medical treatment while living in another country.

**Fig. 1:**
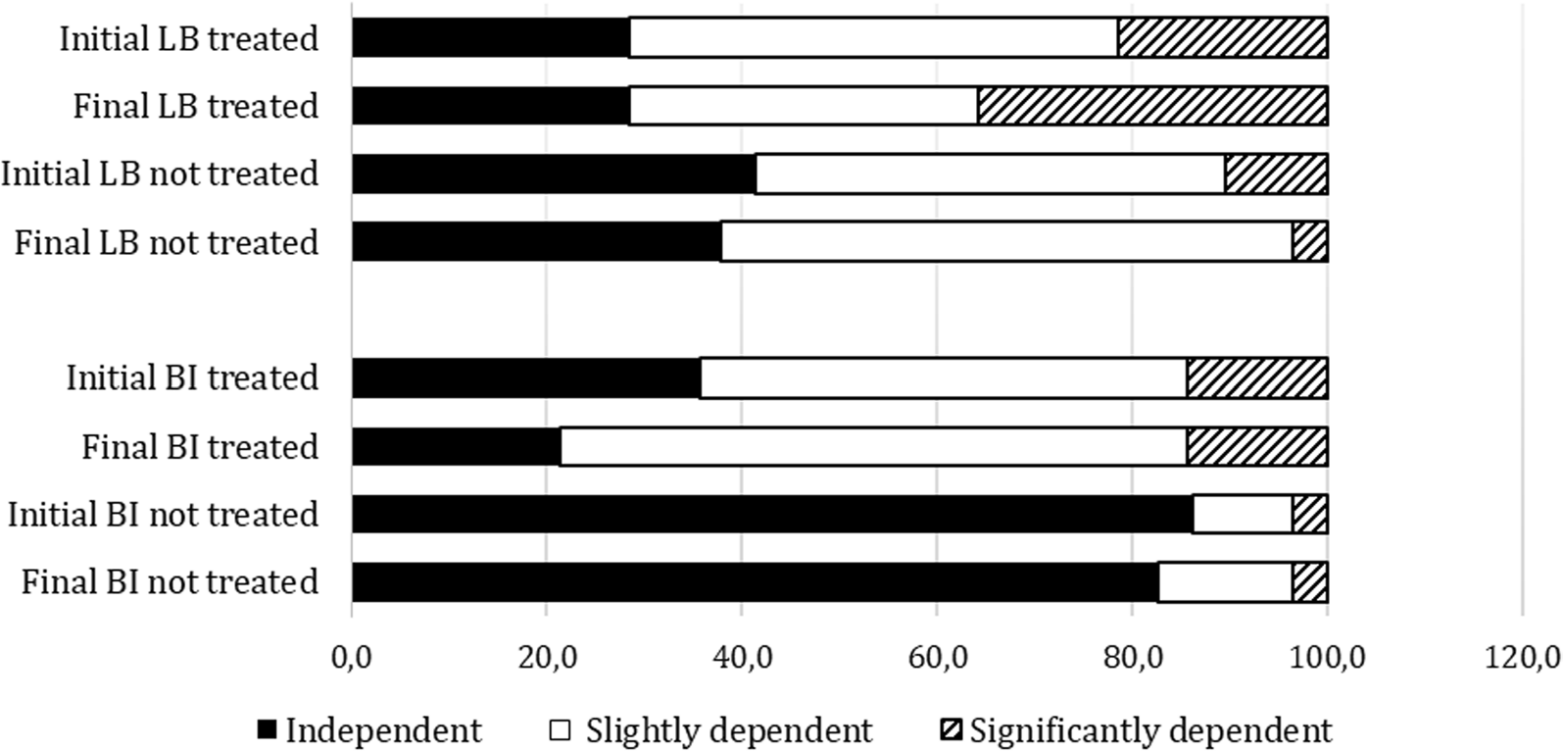
Comparison between untreated and treated patients on the basic (BI) and instrumental (LB) scales

The Barthel Index (BI) and the Lawton and Brody Instrumental Activities of Daily Living (IADL) scale were used to assess patients' physical activity. The results showed that among the patients who participated in the occupational intervention, the majority maintained their initial scores on both scales at six months. However, two subjects (14.3%) lost independence according to the BI. There is a statistically significant relationship between the treatment group and the level of dependency on the BI (*p* < 0.001). No statistically significant differences were found between the treated and untreated groups for IADL. During the 6-month intervention period, these two patients added new co-morbidities to their medical history that had an impact on their basic activities (Fig. 1).

**Fig. 2:**
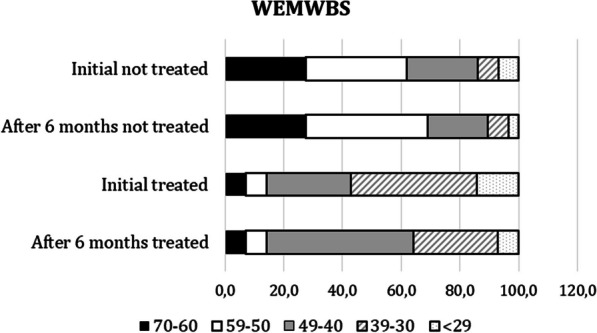
Comparison of scores at baseline and six months post-intervention on the WEMWBS scale

Results from the WEMWBS scale are shown in Fig. 2. During the first semi-structured interview, participants were asked about how their diagnosis affected their psychological wellbeing. Of the 44 participants, 36 (82%) were of the opinion that their mental health had been in some way affected by the diagnosis.

**Fig. 3:**
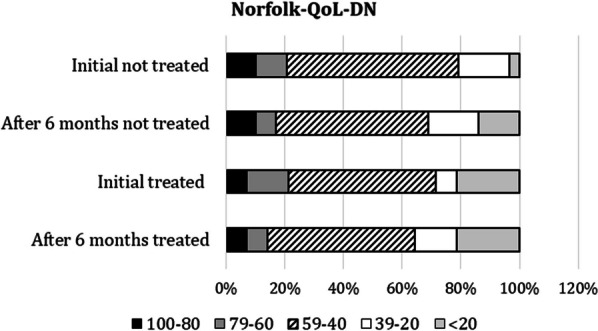
Comparison of Norfolk results. Initial and 6-month data are shown

For the initial WEMWBS scores, the non-treated group had higher scores than the treated group, with most of the treated patients scoring in the 40–70 range, compared to the treated patients who mostly scored in the 49- < 29 range. This suggests that the untreated patients may have had a higher level of mental well-being at baseline (Fig. 3).

After 6 months, both treated and untreated patients showed some improvement in WEMWBS scores. However, the degree of improvement varied between the different score ranges. Treated patients in the 49–60 range improved more than untreated patients in the same range. This suggests that treatment may have had a positive effect on mental well-being. However, it's important to note that treatment may not work for everyone, as some treated patients still experienced a decline in their scores over 6 months. In summary, further statistical analysis is needed to determine the significance of these findings, although the results suggest that treatment had a positive effect on mental well-being.

In the Norfolk QoL-DN questionnaire, all patients (100%) attributed their difficulties with activities of daily living to their ATTRv symptoms, although they continued to perform them to a great extent. The most commonly reported symptoms were pain and loss of sensation in the feet and legs, difficulty with fine motor skills, and fatigue at baseline and 6 months. The Norfolk QoL-DN score in both the treated and non-treated groups appeared to remain stable at 6 months.

Table 1 shows the results of the SF-36 Spanish version v2 questionnaire, which shows that the first occupational approach had the greatest impact on general well-being, physical function and emotional well-being. Quantifying this impact using rating scales and numerical scores proved difficult, although patients expressed in interviews that the diagnosis had a major impact on their daily lives. Nevertheless, significant improvements in limitations related to physical problems and emotional and physical role limitations were observed, and the results remained stable six months later.

**Table 1:** Scores obtained from the SF-36 spanish version v2 questionnnaire at the initial time point and at the 6-month time point

	% of impairment Mean (± SD)			
	Non-Treated		Treated	
	Initial	After 6 months	Initial	After 6 months
Physical functioning	98(23.4)	98 (23.5)	61(20.8)	70 (21.8)
Role limitations from physical problems	45 (47.7)	50 (46.4)	50(30.5)	67 (30.6)
Role limitations from emotional problems	57 (47.6)	57 (47.6)	45 (43.8)	58 (37.5)
Fatigue	98 (23.6)	98 (23.6)	98 (12.8)	98 (13.1)
Emotional well-being	98 (23.1)	98 (23.1)	98 (17.2)	98 (17.2)
Social function	97 (25.8)	99 (27.8)	100 (8.1)	99 (14.1)
Pain	98 (33.4)	100 (33.6)	100 (7.7)	100 (11.9)
General well-being	100 (23.2)	100 (23.2)	95 (10.1)	98(10.5)
Health changes	98 (15.3)	98 (15.3)	98 (9.6)	100(16.1)

Patients expressed awareness that their current situation was likely to worsen as their disease progressed and emphasised the need for interventions to maintain their general wellbeing, highlighting the importance of a multidisciplinary team approach. In terms of occupation, 29% (4 patients) of the 14 study participants took up new hobbies such as Pilates, ceramics and martial arts. 21% (3 patients) enrolled in academic courses and 14% (2 patients) took part in cultural projects. One patient (7%) found a new job. All patients recognised the essential role played by the occupational therapists during the intervention period and expressed the importance of continuing the therapy sessions. They found the project to be a valuable resource for people diagnosed with rare diseases beyond ATTRv due to its flexibility and subjective impact. The continuation of the project as a valuable resource for rare disease patients was agreed by both the patients and the patient advocacy group.

## Discussion

This study describes the implementation of a comprehensive occupational therapy programme to improve activities of daily living and independence in people with a diagnosis of ATTRv. The programme targeted a range of tasks, from basic to more complex activities, with the primary aim of improving patients' overall functioning and quality of life. The results of this landmark research study present interesting aspects that warrant further discussion.

The study was an evaluation of the effectiveness of an occupational therapy programme for people with ATTRv. The results showed that the patients included in the intervention did not show a significant improvement in the basic and instrumental activities of daily living, and their initial level of occupational independence did not improve, especially in the advanced activities. However, patients expressed the importance of continuous and accessible occupational intervention in ATTRv, as it is a degenerative disease, in order to prevent future limitations and maintain the highest level of independence and meaningful occupations after diagnosis. It is worth noting that most of the patients were in Stage I, which allowed them to continue being independent, this could explain the lack of improvement. Although they were aware of the fact that this independence could be compromised over time. Furthermore, for such a psychosocial project, which requires more time and follow-up, the short duration of the study and the follow-up period are a limitation. Although two of the 14 patients experienced comorbidities during the intervention (one patient was in the process of adjusting to treatment for Parkinson's disease, while another had multiple urinary tract infections and complications), both patients remained compliant and refused to stop the occupational intervention.

The study also assessed the impact of the diagnosis on participants' mental wellbeing. The Warwick-Edinburgh Mental Wellbeing Scale and SF-36 spanish verision v2 questionnaire were used. Initially, a change in mental health as a result of the diagnosis was reported by 36 (82%) participants. Thirty-eight (86.3%) still reported psychological distress after six months. However, after six months of intervention, the results showed an improvement in physical and psychological limitations as measured by the SF-36 spanish verision v2 questionnaire. This suggests that the occupational therapy programme was the motivating factor for these positive changes.

An important finding of this study is that despite the short duration of the intervention, in some cases there was a worsening of ATTRv symptoms on the Norfolk scale after the 6-month intervention period. It is unclear whether this was due to disease progression or a lack of meaningful activity and personal decline. This finding is inconclusive due to the small sample size and short intervention period. Further research is needed to evaluate specific interventions targeted at this population. In addition, it should be noted that patients expressed difficulty in numerically measuring mood states or limitations in daily life, as the concept of daily life is not stable and can be difficult to characterise. Nevertheless, the proper implementation of occupational therapy projects in parallel with other psychosocial interventions, such as psychology, and treatments represents an ideal combination to improve patients' quality of life and to improve their quality-of-life occupational balance. More research should also focus on the psychosocial dimension of ATTRv, particularly OT. The inclusion of occupational therapists in multidisciplinary teams is essential for a holistic approach to patient care. Occupational therapy remains underused in health services and in the management of ATTRv, despite the unique benefits of OT. Awareness of occupational needs, integration of occupational interventions and allocation of financial resources to occupational therapy services are hampered by a lack of research on the effectiveness of OT interventions. [11–13]

According to the literature on OT and other diseases, there are significant limitations in this field when it comes to providing appropriate care for patients with rare diseases. Therefore, the development of OT for rare diseases and the provision of appropriate training for occupational therapists is essential. Specific follow-up of diagnosed patients and integration of their professional profile as part of the multidisciplinary team would lead to significant improvements in patients' daily autonomy. This will not only benefit the patient, but also the health care system as a whole by reducing the demands on the patient related to dependency in daily life [38].

This project is the first of its kind to address OT and ATTRv. It highlights the importance of ongoing interventions that not only prolong life expectancy, but also offer a more balanced and meaningful daily life, in conjunction with medical treatments and other psychosocial interventions such as psychology, social work or genetic counselling.

## Limitations

The study had a small sample size. Only 14 of the 44 patients initially interviewed agreed to attend occupational therapy sessions for 6 months. In addition, the generalisability of the findings to this specific patient profile is limited because the majority of patients were in stage I disease. A larger sample size would have allowed for a more comprehensive exploration of the topic, which may have provided additional relevant information.

Given the complexity of the patients' needs and the nature of the therapy offered, the intervention period was relatively short. A longer time frame may have been necessary to fully achieve the study objectives. Nevertheless, the data collected and analysed in this study were likely to be representative and the findings could be a useful starting point for future research. It is worth noting that the lack of established scales, working strategies and trained professionals to deliver effective interventions is a significant limitation in this area. However, despite this challenge, the findings of this study provide a pioneering avenue for occupational therapists to explore further. We can work towards providing more effective services for patients in the future by addressing the limitations of the field and building on the findings of this study.

## Conclusion

The results of the study suggest that a 6-month occupational intervention may not be sufficient to achieve significant and lasting results. This is due to the fluctuating and complex nature of daily life, which cannot be accurately measured by standardised scales unrelated to occupational therapy. In order to optimise outcomes for patients with ATTRv, a specialised occupational programme with a minimum duration of 24 months, personalised follow-up and the involvement of a qualified occupational therapist may lead to improvements in both clinical outcomes and patients' daily lives. This is evidenced by the positive results observed on the SF 36 scale.

Patients expressed an awareness that their current situation is likely to worsen as their disease progresses and highlighted the need for psychosocial interventions to maintain their general wellbeing. This underlines the importance of a real multidisciplinary team approach.

### Supplementary Information

**Additional file 1**. Description of the ATTRv OT intervention.

**Additional file 2**: Description of scales and questionnaires used to evaluate the implementation of the OT program in ATTRv

## Data Availability

The author confirms that all data analysed and generated during this study are included in this published article. The data that support the findings of this study are not openly available due to reasons of sensitivity and are available from the corresponding author upon, ECB, reasonable request. Data are located in controlled access data storage at Hospital Universitario Son Llàtzer.

## Abbreviations

OTA: American occupational therapy association
ATTRv: Hereditary transthyretin amyloidosis
BADL: Basic activities of daily living
BI: Barthel index
CMT: Charcot-marie-tooth disease
IADL: Instrumental activities of daily living
MOHO: Model of human occupation
OT: Occupational therapy
QoL-DN: Quality of life diabetic neuropathy
SF-36: Short-form 36
TTR: Transthyretin
WEMWBS: Warwick-edinburgh mental well-being scale
