# Supplementary material for: Occupational practice in patients with hereditary transthyretin amyloidosis, a qualitative study
Source: Orphanet J Rare Dis. 2023 Nov 10;18:352. doi: 10.1186/s13023-023-02964-3 (PMC10636990; doi:10.1186/s13023-023-02964-3)
Supplement: Supplementary file 1 — Additional file 1. Description of the ATTRv OT intervention. [file 13023_2023_2964_MOESM1_ESM.docx]

**Supplementary 1:** Description of the ATTRv OT intervention.

The Model of Human Occupation (MOHO) is an occupational approach that emphasizes four basic dimensions: occupational motivation, occupational life pattern, subjective performance, and environmental influence [37,38]. This model facilitates a direct relationship between the individual and their environment, which is crucial in determining an appropriate occupational intervention for patients diagnosed with ATTRv. When making occupational choices, the patient assumes an occupational role that requires development, initiating a personal or professional project. MOHO also incorporates the environment and context into occupational intervention, including objects, occupational forms, specific spaces, and/or groups that create a significant context for the individual, and establishes change as a person-centered intervention strategy. As a result, this model equips the patient with the necessary tools to achieve meaningful occupational change [37,38].

**Occupational Intervention**

The occupational therapy program for patients with ATTRv included therapeutic interventions, assessments, and collaborative goal-setting. An initial assessment was conducted at the beginning of the study through a semi-structured interview, which collected personal data (such as name, sex, and age) and qualitative data about the patient's previous psychosocial situation, including their level of autonomy, personal occupations, and employment status. Standardized scales were used to evaluate the patient's autonomy in basic and instrumental activities of daily living, as well as the impact of symptoms on daily living and their mental wellbeing and motivation levels.

Once the initial assessment was completed, therapeutic objectives were defined in consensus with the patient, taking into account their interests and motivations. The treatment plan was then drawn up according to the MOHO model and the AOTA main points (Table 1). The interventions were carried out online, either individually or in groups depending on the patient's specific needs. Sessions were held either weekly or every ten days for 45-50 minutes, and theoretical materials such as informative sheets or videos were provided to allow patients to continue their interventions at home and apply the knowledge in their daily lives.

| **Occupation areas** | Activities of daily living  Education  Work  Leisure time  Social participation |
| --- | --- |
| **Characteristics of the person** | Personal values ​​and beliefs  Body functions  Body structures |
| **Execution skills** | Sensory and perceptual skills  Motor skills and praxis  Emotion regulation skills  Cognitive skills  Communication and social skills |
| **Execution patterns** | Habits  Routines  Roles  Rituals |
| **Contexts and environments** | Cultural  Personal  Physical  Social  Temporary  Virtual |
| **Activity demands** | Necessary objects and their function  Leisure demands  Space demands  Activity sequence and time  Necessary Actions  Required body functions  Required body structures |

**Table 1**. The domains of occupational therapy as described by the American Occupational Therapy Association (AOTA) were utilized in the development of an occupational therapy program for patients with ATTRv.

The occupational therapist implemented the entire OT program, which included various therapeutic interventions:

**Re-education of activities of daily living: BADL (Basic activities of daily living) and IADL (Instrumental activities of daily living).**

The aim of the BADL re-education sessions was to enhance the patient's autonomy by gradually training them in tasks related to mobility and daily living activities. The re-education began with simple activities such as eating and grooming and progressed to functional mobilization sessions focused on IADL. Weekly sessions were conducted to repetitively execute tasks and adjust the activity level according to the patient's capacity and tolerance. The patient's progress in BADL was the first priority, and when they gained autonomy, the focus shifted to IADLs while continuing to motivate and search for meaningful activities. In addition to mobility, fine manual skills, superficial sensitivity, and global limb functionality were also targeted. The sessions included task-oriented exercises and manipulative tasks with repetitive movements to improve manual dexterity, finger grip, strength, and sensitivity. The program also included health education to inform patients about the therapeutic process and empower them to manage their abilities while minimizing associated risks.

**Motivation and meaningful activities.**

During the initial interview, we gathered information on the patients' interests, objectives, and occupational needs in order to address aspects such as motivation and occupational independence. Throughout individual sessions, we utilized a notebook called the "Memory Manual for Amnesia," which allowed patients to write down their goals, thoughts, ideas, and projects without fear of judgment. This notebook served as a form of self-therapy and provided a safe space for patients to express themselves. On a weekly basis, patients were asked to organize their days in the manual and report on the activities they completed or did not complete. This follow-up notebook was essential in helping patients achieve their objectives. In addition, we collaborated with patient advocacy groups to guide patients towards new significant activities such as pilates, theater, new jobs, university degrees, ceramics, religion, cultural events, or martial arts. The patient's empowerment and focus on their role in the therapy process played a critical role in the success of the sessions.

**Advice**

Counseling activities were provided to patients who required support products or compensatory strategies. In accordance with the professional skills of an occupational therapist, the intervention was focused on addressing the physical, psychological, occupational, and social aspects of the person, while considering them as a holistic entity at the center of the intervention. The aim was to support patients in identifying and addressing their needs and challenges, while promoting their overall well-being and functional independence.
