# Supplementary material for: Occupational practice in patients with hereditary transthyretin amyloidosis, a qualitative study
Source: Orphanet J Rare Dis. 2023 Nov 10;18:352. doi: 10.1186/s13023-023-02964-3 (PMC10636990; doi:10.1186/s13023-023-02964-3)
Supplement: Supplementary file 2 — Additional file 2: Description of scales and questionnaires used to evaluate the implementation of the OT program in ATTRv [file 13023_2023_2964_MOESM2_ESM.docx]

**Supplementary Table 1:** Description of scales and questionnaires used to evaluate the implementation of the OT program in ATTRv.

| Barthel Index (BI) | The Barthel Index, or Barthel ADL Index, is a widely used scale for assessing an individual's ability to perform activities of daily living (ADLs). The scale includes 10 items that assess basic self-care activities such as feeding, bathing, grooming, dressing, and toileting, as well as more complex activities such as transferring and mobility. Each item is scored on a 0-3 or 0-5 scale depending on the item, with higher scores indicating greater independence in performing ADLs [26]. |
| --- | --- |
| The Lawton y Brody Scale (IADL) | The Lawton y Brody Instrumental Activities of Daily Living (IADL) Scale is a commonly used assessment tool in healthcare and geriatric settings. the IADL scale measures an individual's ability to perform more complex activities required for independent living such as: shopping, housekeeping or self-care in order to evaluate the ability to live independently.  Each item is scored based on the individual's level of independence, typically using a 3- or 4-point scale. Higher scores indicate greater independence in performing these activities [27]. |
| Warwick-Edinburgh Mental Well-being Scale (WEMWBS) | The Warwick-Edinburgh Mental Well-being Scale (WEMWBS) is a widely used self-report measure of mental well-being. The scale consists of 14 items that assess a range of aspects of well-being, such as positive affect, satisfaction with life, and relationships with others. Participants rate each item on a 5-point scale from "none of the time" to "all of the time". The total score ranges from 14 to 70, with higher scores indicating higher levels of well-being [31]. |
| Norfolk Quality of Life Diabetic Neuropathy (QoL-DN) | The Norfolk QOL-DN is a useful tool for assessing the effectiveness of treatments for diabetic neuropathy and for understanding the impact of the condition on patients' lives. It consists of 35 questions covering domains such as physical functioning, symptoms, emotions, and social functioning. Each question has five possible responses ranging from "no difficulty" to "extreme difficulty” with higher scores indicating worse quality of life [29]. |
| SF-36 spanish version v2 questionnnaire | The SF-36 questionnaire consists of 36 items that assess eight health concepts: physical functioning, role limitations due to physical problems, bodily pain, general health perceptions, vitality, social functioning, role limitations due to emotional problems, and mental health. The scores range from 0 to 100, with higher scores indicating better health-related quality of life [32]. |
